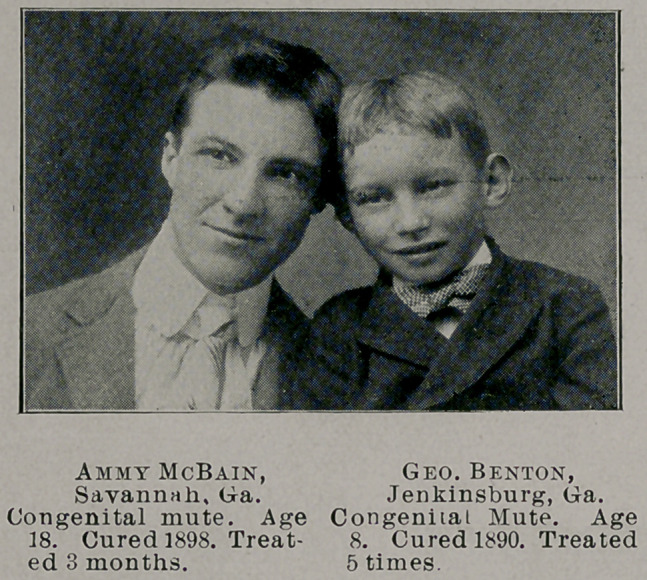# Some Advantages of Stapler’s Rarefier in the Treatment of the Ear

**Published:** 1904-02

**Authors:** Maury M. Stapler

**Affiliations:** Macon, Ga., Member American Medical Association; Member Tri-State Medical Society of Alabama, Georgia and Tennessee; Member Medical Association of Georgia; President of the Macon Medical Society


### SOME ADVANTAGES OF STAPLER'S RAREFIER IN THE TREATMENT OF THE EAR.

BY MAURY M. STAPLER, M.D., MACON, GA.,

Member American Medical Association; Member Tri-State Medical Society of Alabama, Georgia and Tennessee; Member Medical Association of Georgia; President of the Macon Medical Society.

Not until the present time have I felt satisfied that I could offer to the profession a perfected instrument together with a definite procedure surpassing all others in practical results and based upon a new and correct conception of how to apply basic principles to the manipulation and treatment of the ear without endangering the delicate structures of the same.

The instrument consists of a small air-pump and a tubular metal bridge, having five branches, one to be attached to the pump by rubber tubing. Upon the other four branches are fitted rubber tubing, the distal ends of which are fitted air-tight into the nostrils and ears. Those in the ears are to be held in place by a wire support passing over the head, or by an assistant; those in the nostrils by the patient.

The one on the pump is held air-tight in place by the operator in such manner as to be easily removed and used as a speaking-tube to the patient or to allow the passage of air quickly into the cavities when rarefying.

Each branch has a shut-off upon it to be used in making combinations of the branches for special purposes in rarefying.

When inflation is desired the pump is removed and the tubular portion is attached to compressed air tank, and for medication to nebulizing bottles.

STAPLER'S RAREFIER.

No. 1. Correct position for clearing obstruction of the canals and middle ears. Patient swallowing with chin on chest. Correct holding of instrument. All branches of tubing open.

The safety-valve shown in the cut of the instrument has been left off in the later ones because it has been proven that the soft palate will give way under rarefication or inflation and allow the air to pass in time to protect the structures of the ears.

For aseptic reasons it is well to have more than one set of rubber branches to use on the metal tubular bridge.

No. 2. STAPLER'S RAREFIER. Sanitary method of inflating or distending membranes. Nasal branches shut out. Patient in the act of swallowing.

TECHNIC.

See that the cut-offs are open and that the openings of the nasal and aural tips coincide with the canals of these organs and that they are held air-tight with the same. The operator should see that air does not enter at the pump connection.

STAPLER'S RAREFIER.

No. 3. Retracting membranes. Patient swallowing. Aural branches shut out.

Instruct the patient to swallow while the pump is being used. Move the piston with care, that there be no jerk when the palate is engaged. When the ears are being effected the alæ of the nose will be drawn down. Usually there is sufficient leakage of air to allow the alæ to assume the normal as the piston descends in the barrel of the pump. Should this not occur and the pumping is continued,

STAPLER'S RAREFIER.

No. 4. Distending one membrane.

the soft palate will be raised and air will enter. This is the limit of rarefication and I think it necessary only in very obstinate cases and some of the deaf and dumb; although the alæ should be pulled down several times in most cases at each sitting.

Some patients complain of slight pain in the frontal sinus during rarefication and it is, therefore, advisable to proceed with care

STAPLER'S RAREFIER.

No. 5. Retracting one membrane, having aural branch shut out of ear to be retracted.

This is especially necessary when there is a diseased and spongy condition of the mucous lining of the cavities. It is possible to draw blood from the nasal membrane of such cases.

The shut-offs come into use to move the tympanic membranes inward or outward. To effect the first, shut off the aural branches and rarefy through the nasal ones. To retract only one, protect

STAPLER'S RAREFIER.

No. 6. Inflating and applying volatilized medication to all parts of the ears. Patient has on adenoid sack. Hands tied down.

the one not to be effected by putting in the aural branch on that side. To move them outward and overcome adhesion of the membranes to the promontory of the posterior wall of the middle ear, rarefy through the aural branches, the nasal ones being shut-off and the patient should swallow to fill the middle ear with air.

To remove secretions from the mastoid, frontal sinus, lachrymal canals and Autrum of Highmore, protect the tympanic membranes by having all branches open and in place.

After thorough rarefying, the pump is put aside and the tubular portion attached to a compressed air tank for inflation, and for

STAPLER'S RAREFIER.

A—Pump. B—Safety valve. C—For nose. D—For external auditory canals.

medication to nebulizing bottles. Aside from the possibility of drawing a little blood from the nose in cases above mentioned, no untoward effects have followed five years' use of the instrument and method described.

Should the palate be not readily engaged it is due to the fact that the patient is not swallowing, faulty position of the tips in the canals, or leakage of air around the connection.

PROBABLE PHYSIOLOGICAL AND MECHANICAL EFFECTS.

The first effect of rarefying the air in the external auditory canals and middle ears to the same degree at the same time is to hold the tympanic membranes and chain of bone passive in a rarefied chamber.

The normal air-pressure being removed the action of the heart expands the blood-vessels and gives a sense of fullness throughout the mucous surfaces of the head.

The presence of new blood energizes the glandular structure. The flow of mucus acts as a solvent and wash to cleanse the mucous membranes, and aided by the suction current of air the broken-down epithelial cells and microbes are borne away to the eliminating ducts of the body.

Then comes in the compressed air to unfold the creases and empty the hidden pockets and the nebulized medication finds a diseased surface and ramifying into the remotest recesses soothes and cures.

The rich supply of blood-vessels to the interior of the labyrinth probably feels also the effects of removing the normal air-pressure from the round and oval windows and the tympanic membrane and chain of bones being no longer operative.

The force of the heart's action may operate to expand these vessels, which would force the lymph against the membranes of the windows and press them outward and with the membrane of the oval window would move the footplate of the stapes, the connecting chain of bones and the tympanic membranes. Such movement would tend to break down adhesions between the crura of the oval window and the footplate of the stapes and between the stapes, incus and malleus.

Such movement would also stimulate the lymph-glands of the labyrinth and the additional lymph supplied to the labyrinth should support the footplate and chain of bones in their new relations so that there would be no coaptation of raw surfaces and therefore no readhesions.

PRACTICAL RESULTS.

Having the instrument and technic and a knowledge of the conditions to be treated, one should be able to prognosticate for himself those which should be favorably effected.

SIDNEY WEAVER, Montezuma, Ga. Congenital mute. Age 5. Cured Jan. 1, 1904. One treatment.

IRENE WEAVER, Montezuma, Ga. Semi-mute. Greatly improved.

CLIFFORD DAVIS, Stevens Pottery, Ga. Age 5. Cured 1901. Deaf from measles. Cured in 10 days.

HARLOW NASH, Eastman, Ga. Age 4 years. Congenital mute. Hearing and speech established.

KITTIE SMITH, Macon, Ga. Congenital mute. Age 12. Cured 1900. One treatment.

The instrument applies in one way or another to almost all affections of the ears, acute or chronic, and if properly applied ninety per cent. of the ordinary cases in private practice should improve. Excepting those of over thirty-five years of age who have atrophic conditions and the deaf and dumb, fifty out of a hundred should get fifty per cent. of improvement and thirty-five should be entirely restored. These estimates are based upon the opinions of medical men who have observed the technic during the past year.

AMMY MCBAIN, Savannah, Ga. Congenital mute. Age 18. Cured 1898. Treated 3 months.

GEO. BENTON, Jenkinsburg, Ga. Congenital Mute. Age 8. Cured 1890. Treated 5 times.

As to old atrophic conditions I should want more time before making an estimate regarding the benefits to be derived.

DEAF–MUTISM.

Fully appreciating the importance of the statement, with a knowledge of the false hopes it would arouse if untrue, I still assert that there is hope of establishing distinct hearing and speech to all those cases of deaf-mutism in which there is no history of disease involving the nervous system, no manifest deformity and in which the tympanic membranes are not destroyed, be they congenital or adventitious.

With the knowledge acquired in the treatment of this class of cases in the past six years, I should feel disappointed if, with favorable surroundings, I should fail to establish distinct hearing and speech to less than a third of those treated. The treatment consists essentially in what has been related herein before.

Twenty-four cases of all kinds have come to me in the past six years. Of this number one only had more than the rudimentary hearing found in the majority of mutes. This one and nine others were made to hear and talk. Two of these have not kept in touch with me. Eight are still under my supervision and if the testimony of all physicians who have seen them, together with that of their mothers, fathers and friends and the ability on my part to produce them is worth anything, they still have a practical degree of hearing and speech, can repeat any word spoken to them in an ordinary tone of voice, can be called from a distance and use the telephone.

Those demanding better proof should perhaps come and see them. Their ages range from four to eighteen years and the majority of those who came and a majority of those cured were congenital.

### PREVENTION OF DEAFNESS.

Since the eustachian canals of children are shallow and patulous it is an easy matter to remove secretions from the middle ears with the rarefier. It has occurred to my mind that if the secretions were removed every other day from the middle ears in cases of diphtheria and the eruptic fevers, the infecting microbes would be drawn away before inflammatory action could occur. The patient should be in a sitting posture and the rarefication done as already described.

January 5, 1904.

## Figures and Tables

**No. 1. f1:**
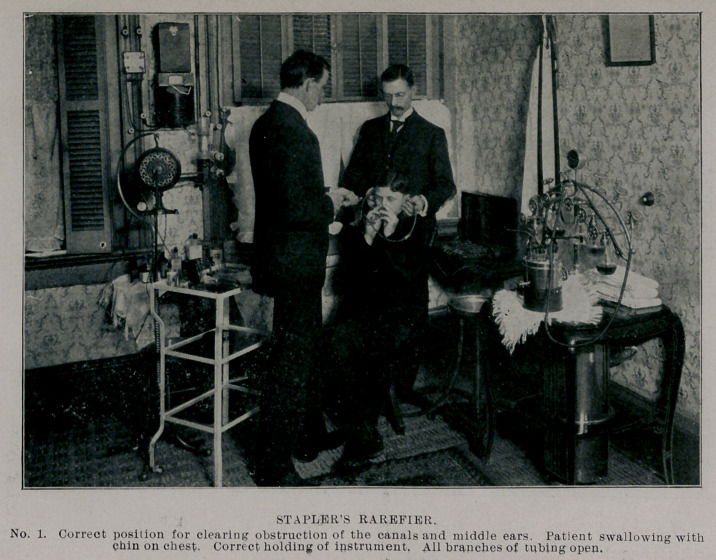


**No. 2. f2:**
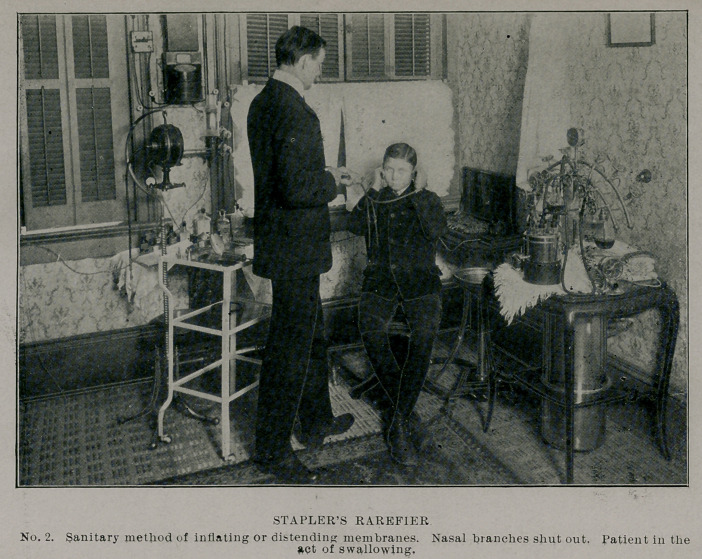


**No. 3. f3:**
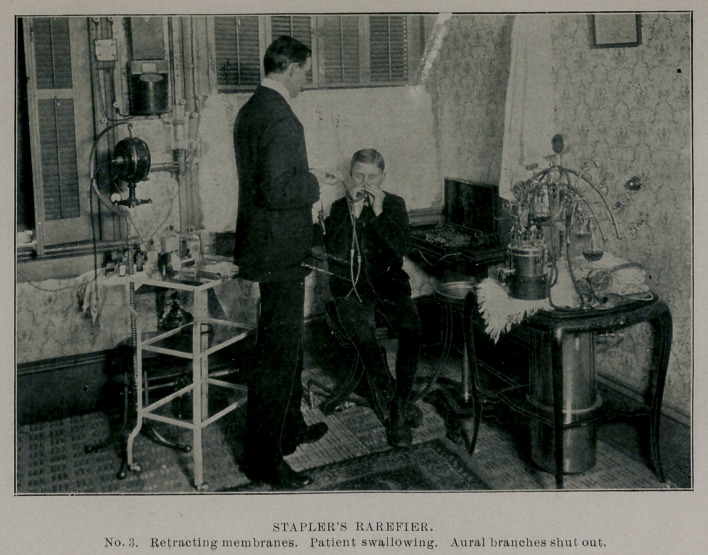


**No. 4. f4:**
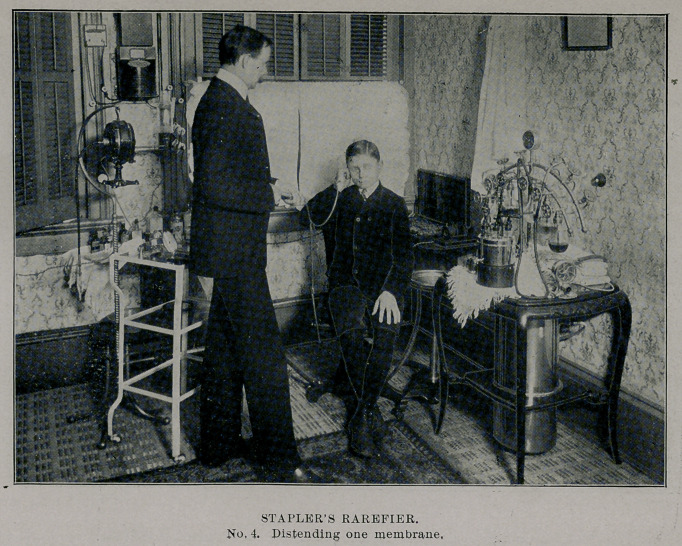


**No. 5. f5:**
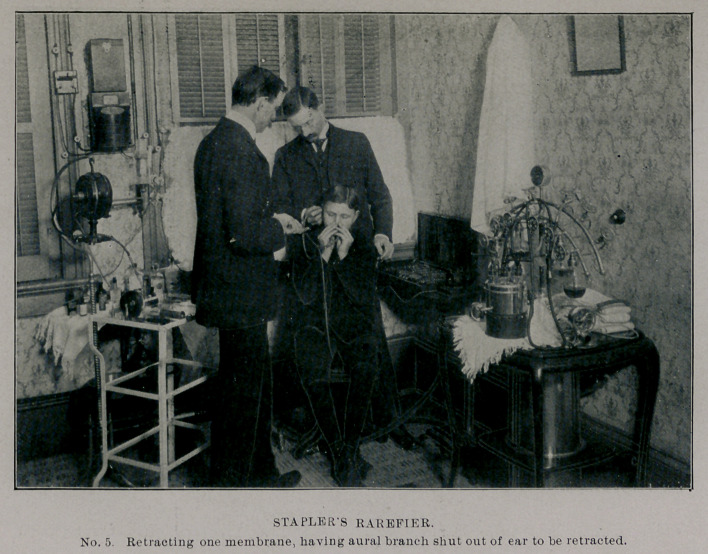


**No. 6. f6:**
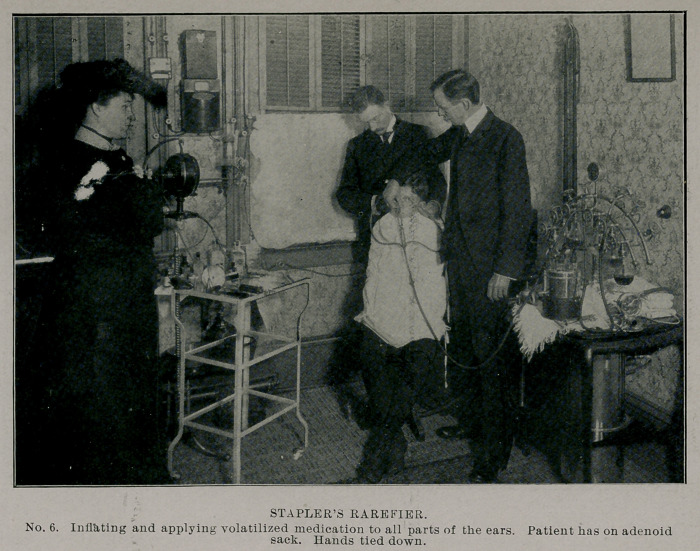


**Figure f7:**
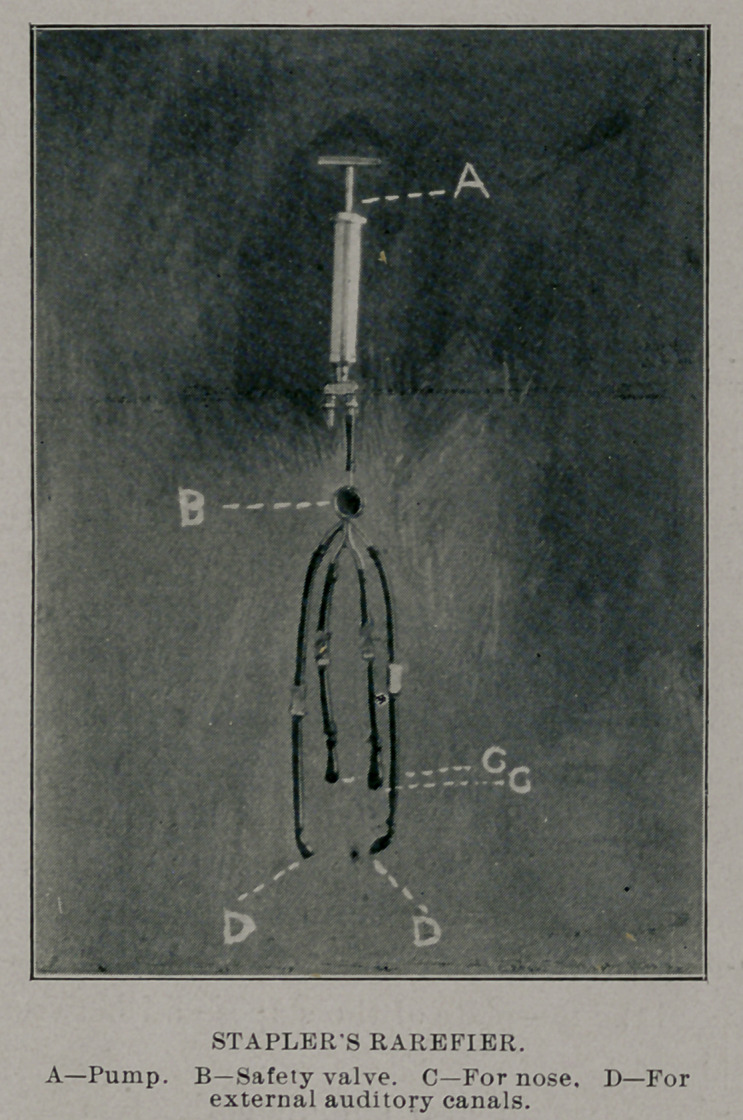


**Figure f8:**
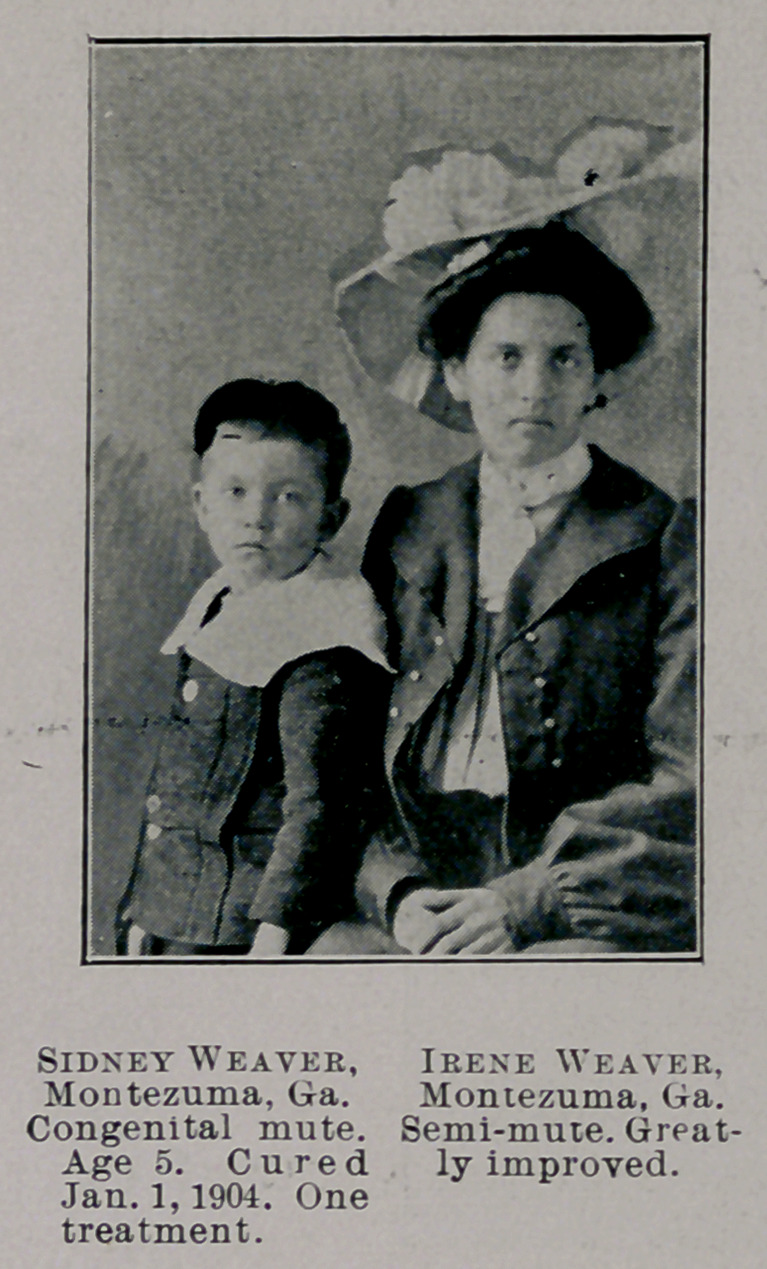


**Figure f9:**
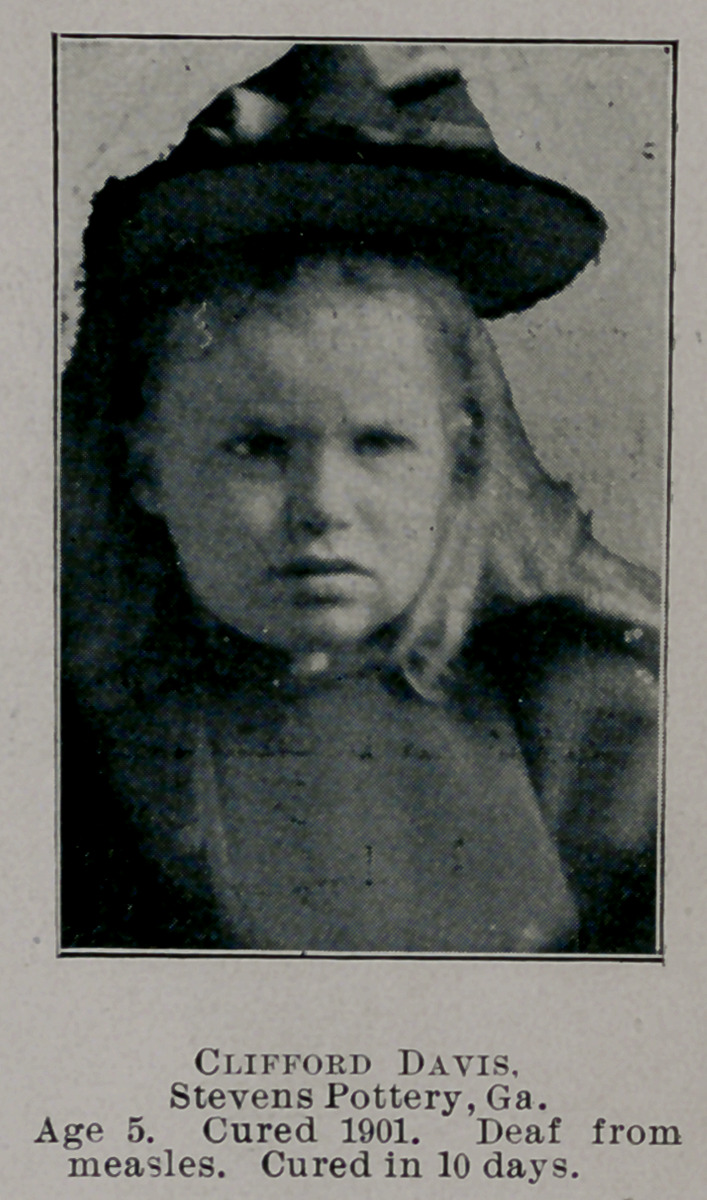


**Figure f10:**
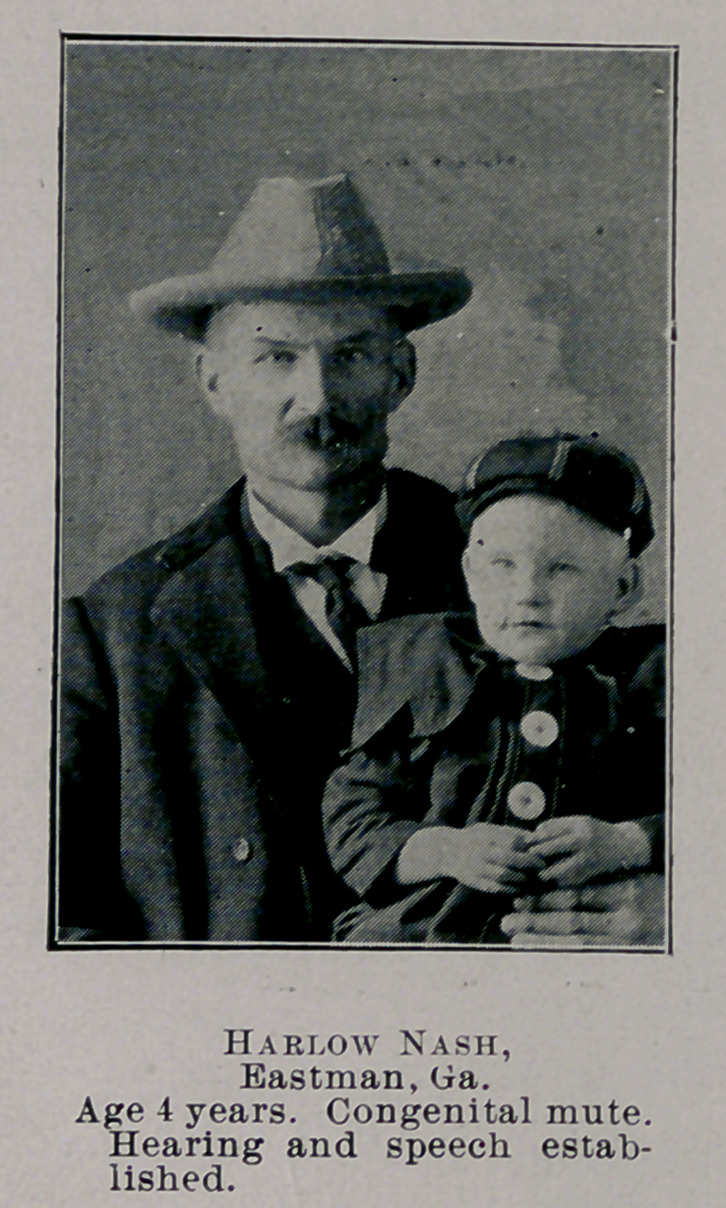


**Figure f11:**
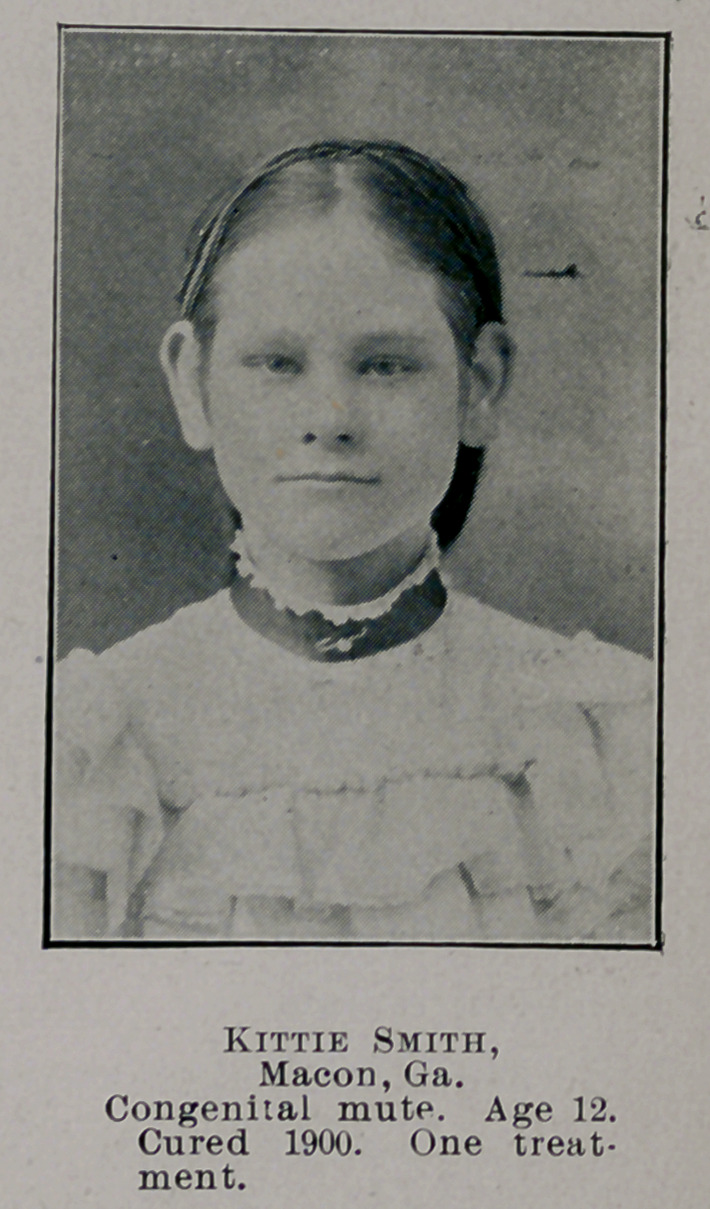


**Figure f12:**